# Determinants of lifestyle behavior change to prevent type 2 diabetes in high-risk individuals

**DOI:** 10.1186/s12966-017-0532-9

**Published:** 2017-06-12

**Authors:** N.R. den Braver, E. de Vet, G. Duijzer, J. ter Beek, S.C. Jansen, G.J. Hiddink, E.J.M. Feskens, A. Haveman-Nies

**Affiliations:** 1 Division of Human Nutrition, Wageningen University & Research, Wageningen, The Netherlands; 2 Department of Epidemiology and Biostatistics, VU Medical Center, Amsterdam Public Health Research Institute, Amsterdam, The Netherlands; 3 Strategic Communication Chair group, Wageningen University & Research, Wageningen, The Netherlands; 4 GGD Noord- en Oost-Gelderland, Warnsveld, The Netherlands

**Keywords:** Type 2 Diabetes Mellitus, Lifestyle intervention, Prevention, Mediation, Behavioral determinants, Primary healthcare

## Abstract

**Background:**

Although there are many effective lifestyle interventions for type 2 diabetes (T2DM) prevention, insight into effective intervention pathways, especially of long-term interventions, is often lacking. This study aims to provide insight into the effective intervention pathways of the SLIMMER diabetes prevention intervention using mediation analyses.

**Methods:**

In total, 240 participants at increased risk of T2DM were included in the analyses over 18 months. The intervention was a combined lifestyle intervention with a dietary and a physical activity (PA) component. The primary and secondary outcomes were change in fasting insulin (pmol/L) and change in body weight (kg) after 18 months, respectively. Firstly, in a multiple mediator model, we investigated whether significant changes in these outcomes were mediated by changes in dietary and PA behavior. Secondly, in multiple single mediator models, we investigated whether changes in dietary and PA behavior were mediated by changes in behavioral determinants and the participants’ psychological profile. The mediation analyses used linear regression models, where significance of indirect effects was calculated with bootstrapping.

**Results:**

The effect of the intervention on decreased fasting insulin was 40% mediated by change in dietary and PA behavior, where dietary behavior was an independent mediator of the association (34%). The effect of the intervention on decreased body weight was 20% mediated by change in dietary and PA behavior, where PA behavior was an independent mediator (17%). The intervention significantly changed intake of fruit, fat from bread spread, and fiber from bread. Change in fruit intake was mediated by change in action control (combination of consciousness, self-control, and effort), motivation, self-efficacy, intention, and skills. Change in fat intake was mediated by change in action control and psychological profile. No mediators could be identified for change in fiber intake. The change in PA behavior was mediated by change in action control, motivation, and psychological profile.

**Conclusion:**

The effect of the SLIMMER intervention on fasting insulin and body weight was mediated by changes in dietary and PA behavior, in distinct ways. These results indicate that changing dietary as well as PA behavior is important in T2DM prevention.

**Electronic supplementary material:**

The online version of this article (doi:10.1186/s12966-017-0532-9) contains supplementary material, which is available to authorized users.

## Background

Lifestyle behaviors have been consistently linked to risk of type 2 Diabetes Mellitus (T2DM) and often targeted in prevention programmes [1]. Although effective lifestyle interventions to prevent T2DM are available, little is known about the behaviors and determinants that mediate intervention effectiveness [2–4]. Moreover, studies reporting intervention effectiveness over the long-term (>12 months) are scarce, especially in studies investigating translation to real-life settings [5]. In order to identify effective targets for T2DM prevention programs that are also promising in the long run, it is important to identify and report effective intervention pathways.

Although changing dietary and physical activity (PA) behaviors is a target of many effective lifestyle programs to reduce T2DM, the effects of dietary intake and physical activity are often poorly understood [1]. Systematic reviews and meta-analyses have attempted to identify effective pathways, however these studies often failed to identify effective pathways through formal mediation analysis [6]. Studies up until now, reviews and meta-analyses have resulted in an understanding that combined lifestyle interventions are often effective [5, 6], while mediation analyses can clarify causal pathways and determinants responsible for intervention success.

One review investigated the mediation of behavioral determinants in the effectiveness of lifestyle interventions in changing behavior and body weight [7]. Motivation, self-efficacy, and self-regulation skills were reported as the most promising mediators of weight change and PA behavior. For dietary intake however, no mediators could be identified because of a lack of clear and consistent evidence [7]. Another meta-analysis of determinants of glycemic control in patients with T2DM indicated PA as a consistent determinant of body mass index (BMI) and self-efficacy as an important determinant of guideline adherence [8].

The SLIMMER (SLIM iMplementation Experience Region Noord- en Oost-Gelderland) diabetes prevention intervention (dietary and PA component) was implemented in Dutch public health and primary healthcare and proved to be effective over the long term (up to 18 months after baseline) [9, 10]. This intervention provides the opportunity to investigate mediation of behaviors and behavioral determinants on the long-term (18 months). In the current study, we firstly investigated whether the associations between the SLIMMER intervention and the outcomes (fasting insulin and body weight) were mediated by changes in dietary behavior and PA behavior. Secondly, we investigated whether the associations between the SLIMMER intervention and specific lifestyle behaviors were mediated by behavioral determinants.

## Methods

### Design and study population

**Fig. 1:**
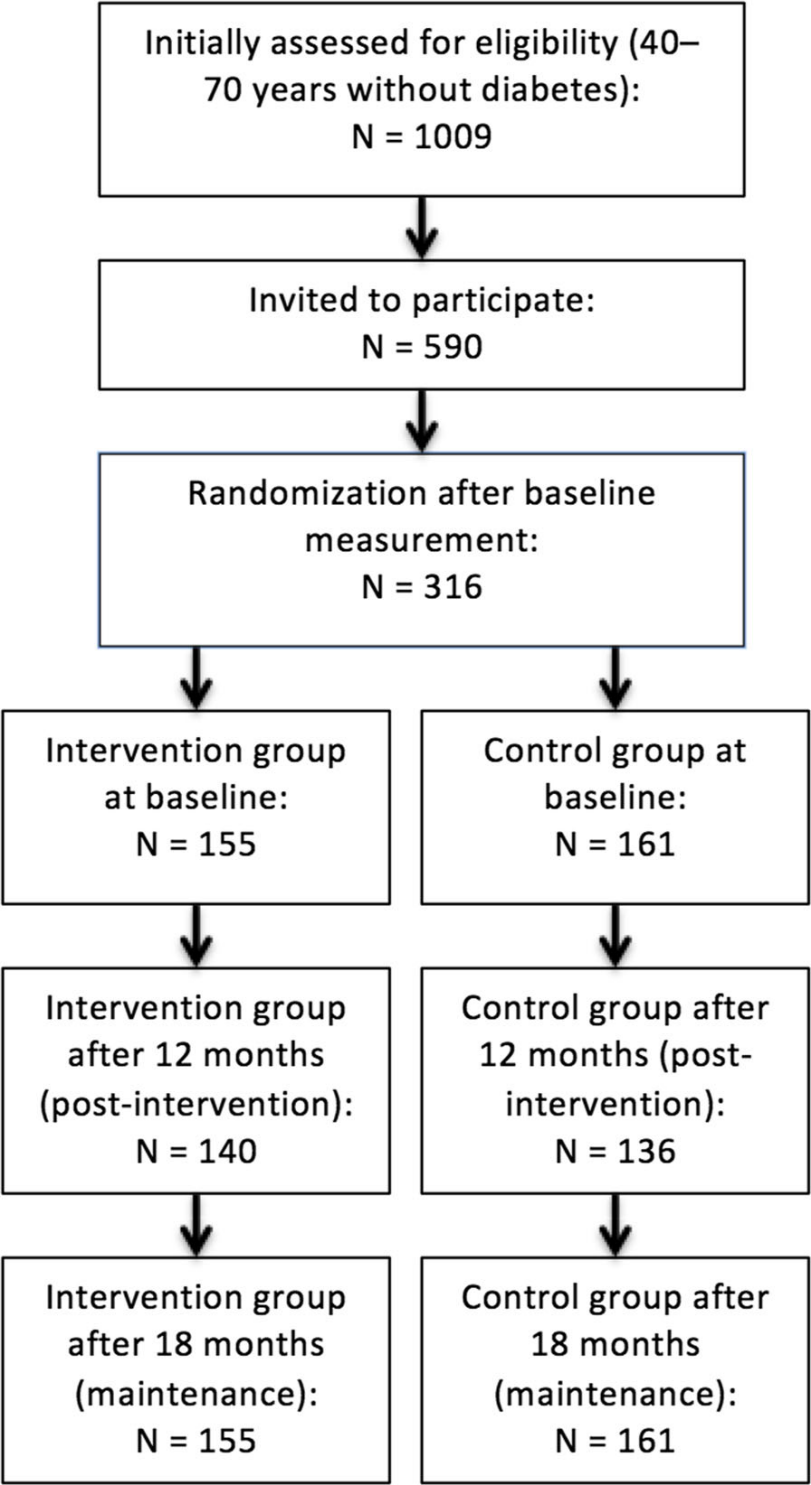
Flow chart

The design and study population of the SLIMMER intervention have been described in detail elsewhere [11, 12]. In short, SLIMMER was a randomized controlled intervention study, and participants were recruited by general practitioners (GPs). Inclusion criteria were (1) aged 40–70 years and (2) impaired fasting glucose (IFG; 6.1–6.9 mmol/L) or a high risk of diabetes (a Diabetes Risk Test score of ≥7 points) [9]. Participants resided in the Dutch cities of Apeldoorn and Doetinchem. In total, 1009 persons were assessed for eligibility. A total of 590 persons were invited to participate, of which 316 participants (response rate 54%) were willing to do so, and 240 had complete follow-up information (Fig. 1). The SLIMMER study was approved by the WU Medical Ethics Committee and participants signed informed consent.

### Intervention

**Fig. 2:**
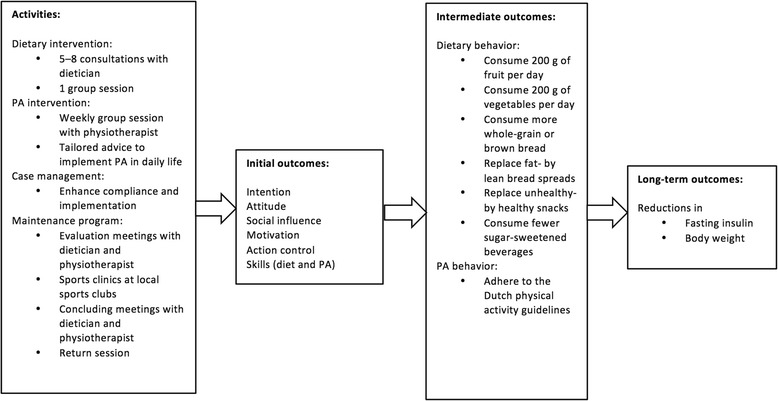
Causal model of SLIMMER intervention

Figure 2 gives an overview of the activities and aims of the SLIMMER intervention. SLIMMER targeted various health outcomes, health behaviors, and behavioral determinants [11, 12]. SLIMMER effectively improved the primary outcome, fasting insulin, and the secondary outcome, body weight, in the short (12 months) and long (18 months) term (other effectively changed health outcomes are reported in an effect paper) [9].

The 10-month SLIMMER intervention consisted of a dietary and a PA component (Fig. 2). In short, the dietary intervention included five to eight consultations with a dietician and one group session. The main goal of the dietary intervention was to promote adherence to the Dutch guidelines for a healthy diet, especially diabetes-related dietary behaviors such as decreasing energy and fat intake and increasing fiber intake [11]. Furthermore, the goal was to achieve 5–10% weight reduction [11]. The dietary intervention is described in more detail elsewhere [11].

The PA intervention consisted of combined aerobic and resistance exercise training, and participants were stimulated to participate at least one hour per week in these group sessions with a physiotherapist [11]. The aim of the PA intervention was to achieve and maintain an active lifestyle, in other words, to adhere to the Dutch physical activity guideline [11].

Throughout the intervention, case management by practice nurses enhanced compliance and implementation [11]. In the last phase of the intervention and three months after its conclusion, the maintenance program was delivered to maintain healthy changes in dietary and PA behaviors [11, 13]. This included sports clinics at local sports clubs, concluding meetings with the dietician and the physiotherapist, and a return session with the PA group, the dietician, and the physiotherapist [11].

### Health outcomes

Health outcomes in this study were similar to those in the overarching SLIMMER study [9]. The primary outcome, fasting insulin (pmol/L), was determined by an oral glucose tolerance test (OGTT) with a glucose load of 75 g, after 10 h of fasting [9]. Fasting insulin was chosen as the primary outcome because of the disease stage that we investigated. Fasting insulin levels may be a more sensitive measure than, for instance, fasting glucose or HbA1c to diagnose impaired fasting glucose [14–16]. The secondary outcome, body weight, was measured with a Tanita BC-418 weight scale to the nearest 0.1 kg. Outcome variables were measured by trained nurses at the research center at baseline (T0), after the end of the intervention (12 months, T1), and six months after the end of the intervention (18 months, T2).

### Assessment of potential mediators

#### Dietary behavior

To assess changes in dietary pattern, six dietary behaviors were targeted in the SLIMMER intervention (Fig. 2), based on Dutch food-based dietary guidelines [11] and common dietary practices in the SLIMMER pilot study [17–19]. The six behaviors were to consume 200 g of fruits and 200 g of vegetables per day, more whole-wheat or brown bread, replace fat-with lean bread spread, replace unhealthy- with healthy snacks, and consume fewer sugar-sweetened beverages (SSB).

Fruit and vegetable consumption were operationalized as grams per day. Percentage fiber from total bread intake was calculated as an indicator of dietary contribution of whole-grain bread. To indicate the fiber content in different types of bread, we performed regression analyses with the difference in % fiber from T0–T2 as outcome and difference in grams per day intake of white bread, brown bread, and whole-grain bread as determinants. Regression coefficients: β_white bread_ = −0.01 (−0.02; −0.004), β_brown bread_ = −0.004 (−0.009; 0.0), β_whole grain bread_ = 0.01 (0.01; 0.02), indicating that increase in whole grain bread was the only coefficient leading to an increase in fiber intake. Proportion of fat intake from total bread spread intake was calculated to indicate the replacement of fat bread spread with lean bread spread. The proportion of total energy intake derived from snacks or SSBs was calculated to express replacement of high-energy snacks or SSBs with low-energy snacks or beverages.

Dietary intake was assessed by a validated, 183-item Food Frequency Questionnaire, with a four-week reference period [20, 21]. A combined score indicated as the nutrition behavior score index (NBS index) was composed, consisting of the six dietary behaviors. The scoring was based on the calculation procedure of the Dutch Healthy Diet Index (DHD index) [22]. The DHD index represented adherence to the Dutch dietary guidelines by nutrient intake, whereas the NBS index represents adherence to the specifically recommended dietary behaviors. Per dietary behavior, the score ranged between 0 and 10, resulting in a total score between 0 (no adherence) and 60 (complete adherence).

#### Physical activity behavior

Physical activity was assessed with the validated Short Questionnaire to Assess Health-enhancing physical activity (SQUASH) [23]. The durations (minutes per week) of total, light, moderate, and vigorous intensity physical activities were calculated. The durations of moderate and vigorous activities were combined to assess compliance with the Dutch PA guidelines; moderate to vigorous-intensity PA (MVPA) for at least 30 min per day on at least five days a week. This variable was operationalized as how often the participant performed MVPA 30 min per week; this could result in a score exceeding the number of days in a week (seven).

#### Behavioral determinants

In order to select behavioral determinants to target in the intervention, six steps were taken:

1. Behavior change techniques (BCTs) used in the SLIMMER intervention were identified.
2. These BCTs were linked to theoretical constructs, as formulated by Bartholomew et al. [24] and Michie et al. [25]. In addition, behavioral determinants from relevant theories, such as the Theory of Planned Behavior [26], and behavioral determinants mentioned as important by healthcare professionals from the SLIMMER pilot study were added.
3. All behavioral determinants were arranged within Michie et al. [25] 14 domains, for which we used a consensus approach in which we discussed the arrangement with experts in the field.
4. A literature search was performed on the relation between these theoretical constructs and specific nutrition and PA behaviors.
5. Items from validated questionnaires and other relevant studies were used.
6. Theoretical constructs for the final questionnaire were chosen based on appearance in the SLIMMER intervention, relation with behaviors supported by the literature, and the availability of items.

The final questionnaire contained items on intention, attitude, social influences, self-efficacy, motivation, action control, and skills.

A questionnaire was developed to measure behavioral determinants of the six dietary behaviors and for PA behavior [11]. Items are based on questions and scales described by Fishbein and Ajzen [26], Lakerveld et al. [2], and Helmink et al. [27]. Each behavioral determinant was measured by several questionnaire items: intention (three items), attitude (six items), social influences (three items), self-efficacy (three items), motivation (two items), action control (three items), dietary skills (five items), and PA skills (two items) (Additional file 1). Each item was rated on a 7 point Likert scale. Cronbach’s alpha was used to check for reliability of the different items belonging to each determinant. A value of ≥0.7 was considered to be acceptable. All sets of items per determinant scored 0.7 or higher, and therefore different items were combined in one score per determinant by taking the mean. In addition to the behavior-specific determinants, a composite measure was computed summing the mean scores of the behavioral determinants for each of the separate behaviors. This composite measure was interpreted as the participant’s psychological profile, a sum score ranging from 7 to 49.

### Statistical analysis

Participants with missing values in the outcome variables, fasting insulin and body weight, at 12 and 18 months were excluded from the analyses. In total, 275 participants were included in the statistical analyses for baseline to 12 months (T0–T1), and 240 were included in the analyses up to 18 months (T0–T2).

Health behaviors that changed significantly after 12 months and were sustained after 18 months, based on previously reported findings [9], were selected for mediation analyses: fruit intake, fiber from bread intake, fat from bread spread intake, and PA. Results after 18 months are reported, and results after 12 months are reported if these differed substantially from the long-term effect.

**Fig. 3:**
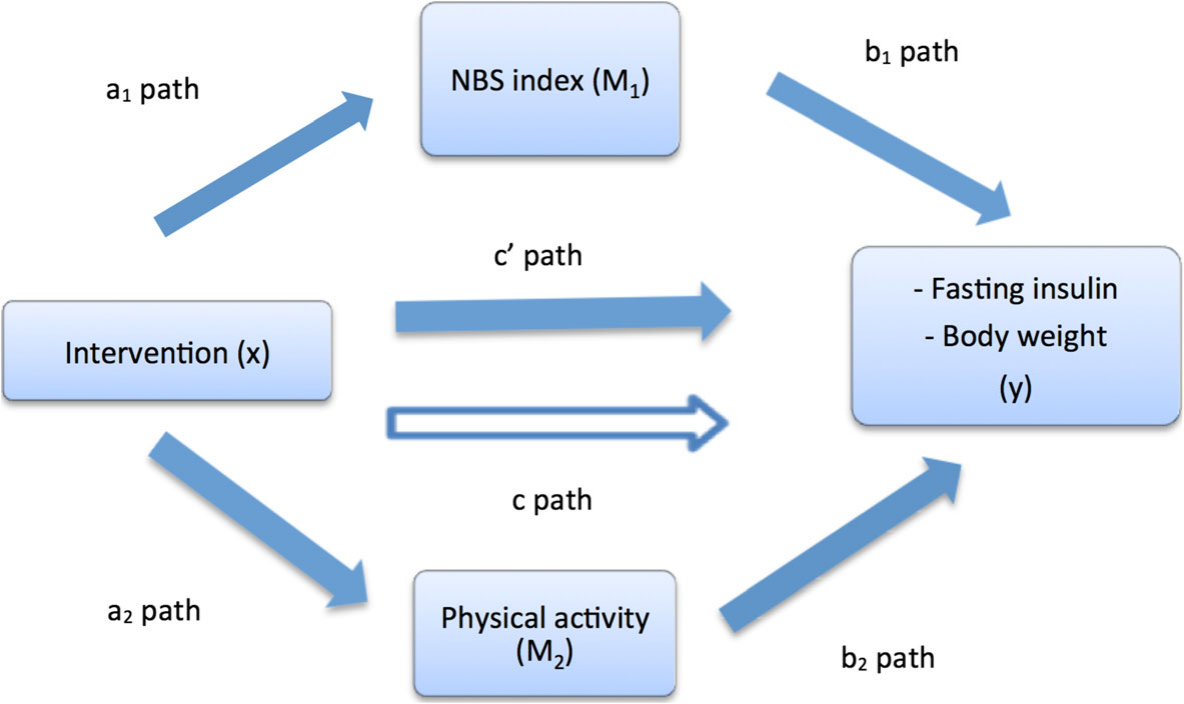
Multiple mediator model for intervention effect via dietary and physical activity behavior. The a_1_ path represents the association between intervention and NBS index. B_1_ represents the association between NBS index and outcome (y), corrected for intervention. A_2_ and b_2_ are interpreted similarly. The c path represents the crude association between intervention and outcome. C′ represents the association between intervention and outcome corrected for NBS and PA

Firstly, it was assessed whether the intervention effect on fasting insulin (pmol/L) and body weight (kg) (y) was mediated by changes in dietary behavior (NBS index) and/or PA behavior (M) in a multiple mediator model (Fig. 3). If mediation effects of dietary and/or PA behavior on fasting insulin could not be determined, we investigated whether these behavior changes showed an effect on decreased fasting insulin via decreased body weight.

**Fig. 4:**
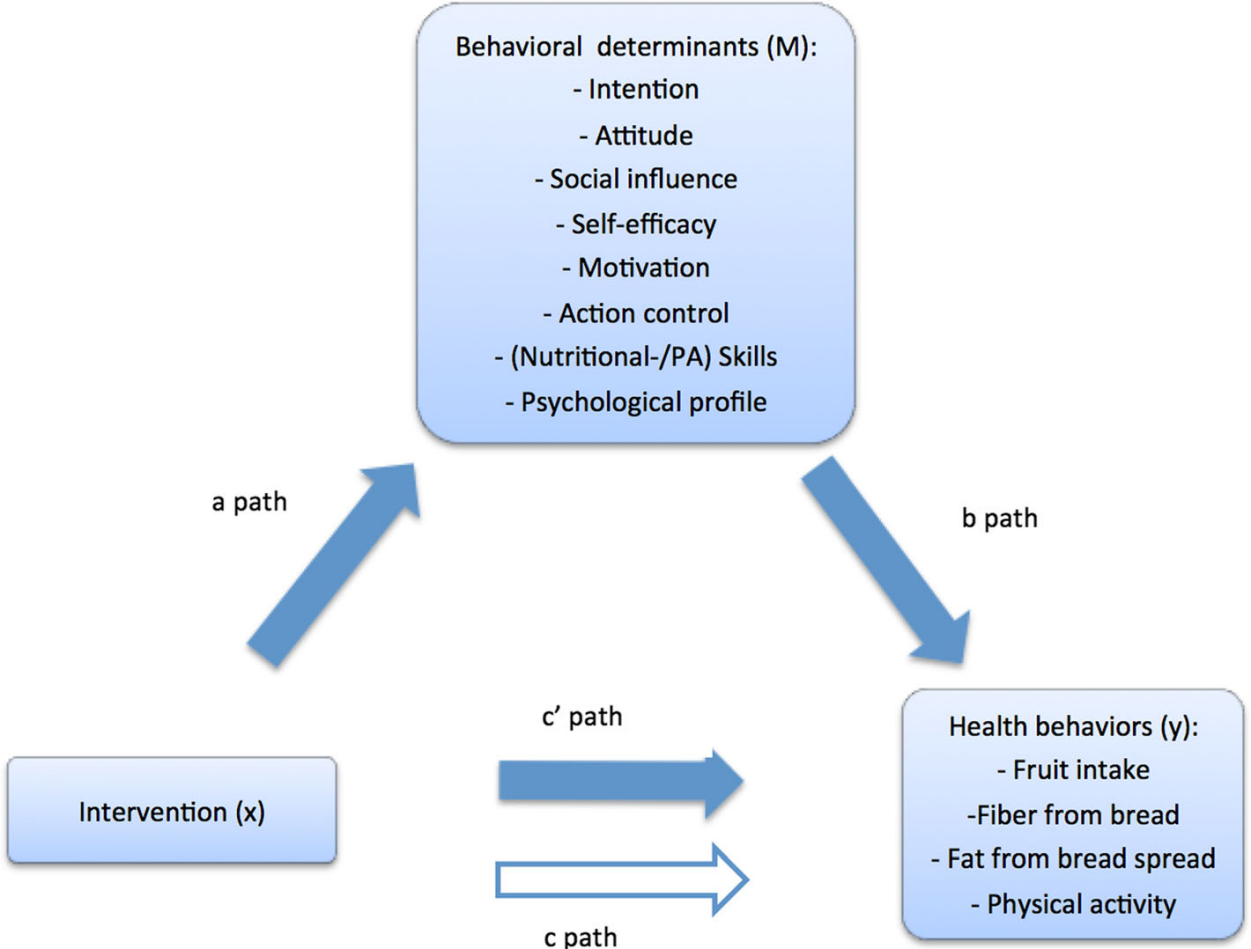
Single mediator models for intervention effect on health behavior (y) via behavioral determinants (m). Path a represents the association between intervention (x) and individual behavioral determinants (m). Path b represents the relation between individual behavior determinants (m) and dietary/PA behaviors (y). C path represents the crude association of the intervention (x) on each of the health behaviors (y). C′ path represents the association between intervention (x) and a health behavior (y) corrected for behavioral determinant (m)

Secondly, it was assessed whether the intervention effect on specific health behaviors (fruit intake, fiber from bread intake, fat from bread spread intake, and PA) was mediated by behavioral determinants in multiple single mediator models (Fig. 4). Behavioral determinants are likely to be correlated, and therefore the assumption of independent mediators in a multiple mediator model was violated. The combined effect of the mediators was alternatively assessed by the psychological profile.

Coefficients were obtained with linear regression analyses. Indirect effects were calculated according to the product-of-coefficients method (a*b) [28]. Standard errors and confidence intervals were calculated with bootstrapping (5000 samples) [29], taking skewed distributions into account. Outcome and mediating variables were adjusted for baseline values in the mediation analyses. Recruitment of subjects took place in three phases due to logistic procedures, therefore all analyses were adjusted for sex and recruitment phase; analyses with fasting insulin were additionally adjusted for medication use. Strict conditions were set for classifying mediating variables in intervention–behavior models to rule out chance findings, given the large number of analyses performed. Variables in these intervention-behavior models were classified as mediators if path a, b, and the indirect path were significant. The direct effect (c’ path) did not have to reduce to zero, because incomplete mediation of the effect is very likely. For significant mediating variables, the proportion mediated was calculated as effect size measure ((a*b)/c). Data were analyzed using the PROCESS macro version 2.13 for SPSS version 22.0 [30].

## Results

### Baseline characteristics

Table 1 and Additional file 2: Table S2 shows the baseline characteristics of participants who completed baseline and first follow-up measurements (*n* = 275). On average, participants were 61 years old with a mean BMI of 30, a mean fasting glucose of 6.6 mmol/L and a mean fasting insulin level of 86.9 pmol/L. The intervention and the control group were similar in terms of baseline characteristics.

**Table 1:** Baseline characteristics of participants presented as mean ± SD or *n* (%) (*n* = 275)

	INT (*n* = 139)	CON (*n* = 136)
Sex (male) *n* (%)	75 (54)	69 (51)
Age	61.1 ± 6.1	61.2 ± 6.6
BMI (kg/m^2^)	30.2 ± 4.5	29.9 ± 4.8
Fasting insulin (pmol/L)	87.8 ± 48.2	86.0 ± 52.8
Fasting glucose (mmol/L)	6.6 ± 0.6	6.6 ± 0.6
HOMA-IR	2.0 ± 1.1	2.0 ± 1.2
*Nutrition:*		
Nutrition behavior score index (0–60)	35.3 ± 9.7	36.6 ± 8.7
Fruit intake (g/dag)	139 ± 120	164 ± 130
Vegetable intake (g/dag)	149 ± 96.6	138 ± 84.7
Fiber intake from total bread (%)	5.6 ± 1.0	5.8 ± 1.2
Fat from total bread spread (%)	21.0 ± 6.4	19.6 ± 6.1
Energy from snack intake (%)	13.4 ± 7.6	15.0 ± 8.3
Energy from SSB intake (%)	2.8 ± 3.3	2.3 ± 2.7
*Physical activity:*		
MVPA (# times, 30 min per week)	9.0 ± 5.5	9.8 ± 5.3
Moderate PA (min/week)	593 ± 692	559 ± 552
Vigorous PA (min/week)	354 ± 427	417 ± 450
*Psychological profile:*		
Fruit intake	40.2 ± 6.6	40.0 ± 6.3
Vegetable intake	41.0 ± 5.7	40.5 ± 6.0
Whole-grain and brown bread intake	41.4 ± 6.1	40.5 ± 6.9
Lean bread spread intake	40.1 ± 5.5	39.2 ± 5.8
Healthy snacks	39.5 ± 5.1	38.7 ± 5.2
SSB intake	40.6 ± 6.3	40.6 ± 5.7
Physical activity	40.4 ± 5.7	40.2 ± 5.7

*HOMA-IR* Homeostasis Assessment Model for Insulin Resistance, *MVPA* Moderate- to vigorous-intensity physical activity, *PA* Physical acitivity, *SSB* Sugar sweetened beverages

### Mediation analyses

Table 2 presents the mediation of change in health behavior between the intervention and health outcomes. The intervention effect on decreased fasting insulin was 40% mediated by dietary and PA behavior together in the multiple mediator model (Table 2). The NBS score accounted significantly for 34% mediation of the association between the intervention and fasting insulin. PA was not a significant mediator of the association.

**Table 2:** Mediation of health behavior change between intervention and health outcomes (18 months, *n* = 240)

**T0–T2** **Fasting insulin (pmol/L)**	**X -- > Y (c path)** ^**b,i**^								
	*B(SE)*		*95%-CI*						
Crude analysis	−6.8 (3.4)		−7.2; −6.4						
	**X -- > Y (c’ path)** ^**c,i**^		**X -- > M (a path)** ^**d,i**^		**M -- > Y (b path)** ^**e,i**^		Indirect effect (a*b) ^**f**^		**Proportion mediated** ^**g**^
	*B (SE)*	*95%-CI*	*B (SE)*	*95%-CI*	*B (SE)*	*95%-CI*	*B (SE)*	*95%-CI* ^*h*^	(a*b)/c
NBS index^a^			4.9 (0.9)	3.2; 6.7	−0.5 (0.3)	−1.1; 0.1	−2.4 (1.4)	−5.3; 0.0	0.34
MVPA^a^			1.6 (0.5)	0.6; 2.7	−0.3 (0.5)	−1.2; 0.7	−0.4 (0.9)	−2.6; 1.0	-
Combined	−4.3 (4.2)	−12.7; 4.0					−2.9 (1.6)	−6.3; −0.1	0.40
**T0–T2** **Body weight (kg)**	**X -- > Y (c path)** ^**b,h**^								
	*B(SE)*	*95%-CI*							
Crude analysis	−2.4 (0.6)	−3.5; −1.3							
	**X -- > Y (c’ path)** ^**c,h**^		**X -- > M (a path)** ^**d,h**^		**M -- > Y (b path)** ^**e,h**^		Indirect effect (a*b) ^**f**^		**Proportion mediated** ^**g**^
	*B (SE)*	*95%-CI*	*B (SE)*	*95%-CI*	*B (SE)*		*B (SE)*	*95%-CI* ^*h*^	*B (SE)*
NBS index^a^			4.9 (0.9)	3.2; 6.7	0.0 (0.04)	−0.1; 0.1	−0.1 (0.2)	−0.4; 0.3	-
MVPA^a^			1.6(0.5)	0.6; 2.7	−0.2 (0.1)	−0.4; −0.1	−0.4 (0.2)	−0.9; −0.1	0.17
Combined	−1.9 (0.6)	−3.1; −0.8					−0.5 (0.3)	−1.0; 0.0	0.20

^a^NBS index defined as a score ranging from 0 to 60, MVPA (# times, 30 min per week)

^b^C path (total effect): the association between intervention and outcomes (fasting insulin and body weight). All analyses are adjusted for baseline value, sex, recruitment phase, and medication use

^c^C’ path (direct effect): the association between intervention and outcomes, adjusted for mediators (NBS index and PA)

^d^A path: association between intervention and NBS index or PA

^e^B path: association between NBS index or PA and outcomes

^f^Indirect effect (a*b): the indirect effect of the intervention on outcome through NBS index and/or PA

^g^Proportion effect mediated ((a*b)/c): the proportion of the total effect that was mediated through NBS index and or PA

^h^Standard error and confidence interval for indirect effects were calculated with bootstrapping (5000 samples)

^i^All analyses use linear regression models

The intervention was associated with a 2.4 kg (95%-CI: -3.5; −1.3) decrease in body weight after 18 months. This association was 20% mediated by dietary and PA behavior together (Table 2).

Nutrition was not a significant mediator, whereas PA significantly accounted for 17% of the association.

Additional analyses showed that the association between PA behavior and fasting insulin was significantly mediated by body weight (β_direct_ = 0.14 (−0.8; 1.12), β_indirect_ = −0.9 (−1.5; −0.4)).

Table 3 presents the mediation of change in behavioral determinants between the intervention and dietary behaviors. The intervention was associated with a 32.3 g/day (95%-CI: 6.3; 58.4) increase in fruit intake after 18 months. This association was 62% mediated by the participants’ psychological profile (Table 3). Behavioral determinants that mediated individually in this association were action control (63%), motivation (51%), self-efficacy (35%), intention (35%), and dietary skills (12%). Attitude made a significant 43% contribution only in the long term after 18 months, but not after 12 months. Social influence did not mediate the association between the intervention and fruit intake.

**Table 3:** Mediation of change in behavioral determinants between intervention and dietary behavior change (18 months, *n* = 240)

**T0–T2 Fruit intake (g/day)**	**X -- > Y (c path)** ^**a,h**^								
	*B(SE)*		*95%-CI*						
Crude analysis	32.3 (13.2)		6.3; 58.4						
	**X -- > Y (c’ path) direct** ^**b,h**^		**X -- > M (a path)** ^**c,h**^		**M -- > Y (b path)** ^**d,h**^		Indirect effect (a*b) ^**e,g**^		**Proportion mediated** ^**f**^
	*B (SE)*	*95%-CI*	*B (SE)*	*95%-CI*	*B (SE)*	*95%-CI*	*B (SE)*	*95%-CI*	*B (SE)*
Intention	20.3 (12.6)	−4.5; 45.2	0.4 (0.2)	0.1; 0.7	26.7 (5.0)	16.8; 36.5	10.9 (5.1)	2.9; 23.6	0.35
Attitude	16.0 (12.7)	−9.1; 41.1	0.6 (0.1)	0.1; 0.6	33.6 (6.7)	20.4; 46.7	11.9 (4.5)	4.7; 22.8	0.43
Social influence	29.4 (13.3)	3.2; 55.6	0.2 (0.1)	−0.1; 0.5	10.8 (6.0)	−1.0; 22.7	2.2 (2.1)	−0.4; 9.0	-
Self-efficacy	21.0 (12.4)	−3.5; 45.5	0.3 (0.1)	0.1; 0.6	34.7 (5.8)	23.3; 46.2	11.0 (5.3)	1.3; 22.7	0.35
Motivation	15.0 (11.7)	−8.0; 38.0	0.5 (0.2)	0.1; 0.9	31.3 (3.6)	24.1; 38.5	15.9 (6.6)	3.4; 29.1	0.51
Action control	11.4 (11.8)	−11.8; 34.6	0.4 (0.2)	0.2; 0.7	43.0 (5.2)	32.7; 53.3	19.0 (6.9)	6.3; 33.8	0.63
Dietary skills	28.2 (13.5)	1.6; 54.8	0.3 (0.1)	0.1; 0.5	15.3 (8.4)	−1.3; 31.9	3.9 (2.7)	0.3; 11.5	0.12
Psychological profile	11.1 (12.2)	−13.0; 35.2	2.5 (0.8)	1.0; 4.1	7.0 (1.0)	5.0; 9.0	17.7 (5.8)	6.7; 29.7	0.62
**T0–T2 Fiber intake from bread (%)**	**X -- > Y (c path)** ^**a, h**^								
	*B(SE)*		*95%-CI*						
Crude analysis	0.3 (0.2)		0.0; 0.6						
	**X -- > Y (c’ path) direct** ^**b,h**^		**X -- > M (a path)** ^**c,h**^		**M -- > Y (b path)** ^**d,h**^		Indirect effect (a*b) ^**e,g**^		**Proportion mediated** ^**f**^
	*B (SE)*	*95%-CI*	*B (SE)*	*95%-CI*	*B (SE)*		*B (SE)*	*95%-CI*	*B (SE)*
Intention	0.3 (0.2)	0.0; 0.6	0.4 (0.2)	−0.1; 0.8	0.0 (0.0)	−0.1; 0.1	0.0 (0.0)	−0.1; 0.0	-
Attitude	0.3 (0.2)	0.0; 0.6	0.0 (0.2)	−0.3; 0.3	0.1 (0.1)	−0.1; 0.2	0.0 (0.0)	0.0; 0.0	-
Social influence	0.3 (0.2)	0.0; 0.6	0.1 (0.2)	−0.2; 0.4	−0.1 (0.1)	−0.2; 0.1	0.0 (0.0)	−0.1; 0.0	-
Self-efficacy	0.3 (0.2)	0.0; 0.6	0.1 (0.2)	−0.2; 0.4	0.0 (0.1)	−0.1; 0.2	0.0 (0.0)	0.0; 0.0	-
Motivation	0.3 (0.2)	0.0; 0.6	0.0 (0.2)	−0.3; 0.4	−0.1 (0.1)	−0.2; 0.0	0.0 (0.0)	−0.1; 0.0	-
Action control	0.3 (0.2)	0.0; 0.6	0.2 (0.1)	−0.1; 0.4	0.1 (0.1)	0.0; 0.3	0.0 (0.0)	0.0; 0.1	-
Dietary skills	0.3 (0.2)	0.0; 0.6	0.3 (0.1)	0.1; 0.5	0.1 (0.1)	−0.1; 0.3	0.0 (0.0)	0.0; 0.1	-
Psychological profile	0.3 (0.2)	0.0; 0.6	0.9 (0.8)	−0.6; 2.4	0.0 (0.0)	0.0; 0.0	0.0 (0.03)	0.0; 0.1	-
**T0–T2 Fat intake from bread spread (%)**	**X -- > Y (c path)** ^**a,h**^								
	*B(SE)*		*95%-CI*						
Crude analysis	−2.6 (0.8)		−4.2; −1.1						
	**X -- > Y (c’ path) direct** ^**b,h**^		**X -- > M (a path)** ^**c,h**^		**M -- > Y (b path)** ^**d,h**^		Indirect effect (a*b) ^**e,g**^		**Proportion mediated** ^**f**^
	*B (SE)*	*95%-CI*	*B (SE)*	*95%-CI*	*B (SE)*	*95%-CI*	*B (SE)*	*95%-CI* ^*g*^	(a*b)/c
Intention	−2.4 (0.8)	−4.0; −0.9	0.3 (0.2)	0.0; 0.7	−0.6 (0.3)	−1.2; 0.0	−0.2 (0.1)	−0.6; 0.0	-
Attitude	−2.6 (0.8)	−4.1; −1.1	0.1 (0.1)	−0.1; 0.4	−0.8 (0.4)	−1.6; 0.0	−0.1 (0.1)	−0.5; 0.1	-
Social influence	−2.6 (0.8)	−4.1; −1.0	0.4 (0.2)	0.1; 0.7	−0.5 (0.3)	−1.2; 0.2	−0.2 (0.1)	−0.6; 0.0	-
Self-efficacy	−2.5 (0.8)	−4.0; −1.0	0.1 (0.1)	−0.2; 0.4	−1.0 (0.4)	−1.7; −0.2	−0.1 (0.2)	−0.5; 0.2	-
Motivation	−2.5 (0.8)	−4.0; −1.0	0.2 (0.2)	−0.2; 0.5	−0.9 (0.3)	−1.5; −0.3	−0.2 (0.2)	−0.7; 0.1	-
Action control	−2.2 (0.8)	−3.7; −0.7	0.3 (0.1)	0.1; 0.6	−1.2 (0.4)	−1.9; −0.5	−0.4 (0.2)	−1.0; −0.1	0.15
Dietary skills	−2.5 (0.8)	−4.0; −1.0	0.3 (0.1)	0.1; 0.5	−0.2 (0.5)	−1.2; 0.8	−0.1 (0.2)	−0.5; 0.2	-
Psychological profile	−2.2 (0.8)	−3.7; −0.7	1.7 (0.7)	0.3; 3.1	−0.2 (0.1)	−0.4; −0.1	−0.4 (0.2)	−1.0; −0.1	0.15

^a^C path (total effect): the association between intervention and dietary behaviors (fruit intake, fiber intake from bread, fat intake from bread spread). All analyses are adjusted for baseline value, sex, and recruitment phase

^b^C’ path (direct effect): the association between intervention and dietary behaviors, additionally adjusted for mediator (behavioral determinants)

^c^A path: association between intervention and behavioral determinant

^d^B path: association between behavioral determinant and dietary behavior

^e^Indirect effect (a*b): the indirect effect of the intervention on dietary behavior through behavioral determinant

^f^Proportion effect mediated ((a*b)/c): the proportion of the total effect that was mediated through behavioral determinant

^g^Standard error and confidence interval for indirect effects were calculated with bootstrapping (5000 samples)

^h^All analyses used linear regression models

The intervention was associated with a 0.3% (95%-CI: 0.0; 0.6) increase in fiber intake from bread after 18 months. No behavioral determinants could be identified as mediators in this association (Table 3). After 12 months, the intervention was associated with a 0.3% (95%-CI: 0.0; 0.5) increase in fiber intake from bread, and only dietary skills explained 14% of this association. None of the other behavioral determinants mediated the relation between the intervention and fiber intake.

The intervention was associated with a 2.6% (95%-CI: -4.2; −1.1) decrease in fat intake from bread spread, after 18 months. This association was 15% mediated by the participants’ psychological profile after 18 months (Table 3). Action control was the only individual determinant that significantly mediated the association, explaining 15%.

The intervention was associated with an increase in physical activity of 1.6 (95%-CI: 0.6; 2.7) times 30 min of MVPA per week, after 18 months. This association was 17% mediated by the participants’ psychological profile (Table 4). Action control (19%) was a significantly mediating variable. Intention, attitude, social influence, self-efficacy, motivation, and PA skills did not mediate the association between the intervention and PA.

**Table 4:** Mediation of change in behavioral determinants between intervention and MVPA change (18 months, *n* = 240)

**T0–T2** **MVPA (# times, 30 min per week)**	**X -- > Y (c path)** ^**a,h**^								
	***B(SE)***	***95%-CI***							
Crude analysis	1.7 (0.5)	0.6; 2.7							
	**X -- > Y (c’ path) direct** ^**b,h**^		**X -- > M (a path)** ^**c,h**^		**M -- > Y (b path)** ^**d,h**^		Indirect effect (a*b) ^**e,g**^		**Proportion mediated** ^**f**^
	***B (SE)***	***95%-CI***	***B (SE)***	***95%-CI***	***B (SE)***	***B (SE)***	***95%-CI***	***B (SE)***	***95%-CI***
Intention	1.6 (0.5)	0.5; 2.6	0.2 (0.2)	−0.1; 0.5	0.5 (0.2)	0.0; 0.9	0.1 (0.1)	0.0; 0.4	-
Attitude	1.5 (0.5)	0.4; 2.5	0.2 (0.1)	−0.1; 0.4	0.5 (0.3)	−0.02; 1.1	0.1 (0.1)	0.0; 0.4	-
Social influence	1.6 (0.5)	0.6; 2.7	0.2 (0.1)	0.0; 0.5	0.1 (0.3)	−0.5; 0.6	0.0 (0.1)	−0.1; 0.2	-
Self-efficacy	1.5 (0.5)	0.4; 2.5	0.2 (0.1)	0.0; 0.5	0.7 (0.3)	0.1; 1.	0.2 (0.1)	0.0; 0.5	-
Motivation	1.4 (0.5)	0.4; 2.5	0.3 (0.2)	0.0; 0.6	0.8 (0.2)	0.4; 1.2	0.2 (0.1)	0.0; 0.6	-
Action control	1.3 (0.5)	0.3; 2.4	0.4 (0.1)	0.1; 0.7	0.8 (0.2)	0.3; 1.2	0.3 (0.1)	0.1; 0.7	0.19
Physical activity skills	1.6 (0.5)	0.5; 2.6	0.3 (0.2)	0.0; 0.6	0.3 (0.2)	−0.1; 0.8	0.1 (0.1)	0.0; 0.4	-
Psychological profile	1.4 (0.5)	0.3; 2.4	1.9 (0.7)	0.5; 3.3	0.1 (0.1)	0.1; 0.2	0.3 (0.1)	0.1; 0.6	0.17

^a^C path (total effect): the crude association between intervention and PA behavior

^b^C’ path (direct effect): the association between intervention and PA behavior, adjusted for mediator (behavioral determinants)

^c^A path: association between intervention and behavioral determinant

^d^B path: association between behavioral determinant and PA behavior

^e^Indirect effect (a*b): the indirect effect of the intervention on PA behavior through behavioral determinant

^f^Proportion effect mediated ((a*b)/c): the proportion of the total effect that was mediated through behavioral determinant

^g^Standard error and confidence interval for indirect effects were calculated with bootstrapping (5000 samples)

^h^All analyses used linear regression models

## Discussion

The present study found firstly that dietary and PA behavior together are important mediators in the association between the SLIMMER intervention and fasting insulin and body weight. Diet was an individual mediator of the association between the intervention and fasting insulin, whereas MVPA was an individual mediator in the association between the intervention and body weight.

Systematic reviews and meta-analyses have observed combined lifestyle interventions to be effective in reducing T2DM risk, while long-term effective studies and translations to real-life settings were scarce [1, 5, 6]. The effective SLIMMER intervention provided the opportunity to test mediating effects of lifestyle behaviors and behavioral determinants toward long-term intervention effectiveness in a real-life setting and sheds light on the different working mechanisms.

Previously, mixed results have been reported regarding the role of PA behavior in lifestyle interventions. Most studies have shown that diet and PA interventions are best combined to achieve changes in glycemic measures, rather than a PA intervention only [1, 31, 32]. The present study is in line with this and provides additional information on the causal path. In the present study increased MVPA was observed to be an individual mediator for weight loss, and weight loss in turn was a mediator between increased MVPA and reduced fasting insulin. A recent meta-analysis confirmed the importance of a combination of a PA intervention and a weight loss program in the prevention of T2DM [33]. MVPA therefore seems to reduce fasting insulin via weight loss, whereas diet seems to have a more direct role in reducing fasting insulin. The finding that diet seems to have a more direct influence on reduction in fasting insulin, than MVPA, could be biologically explained by the associations found in earlier studies between fiber and fat intake with glucose intolerance [34].

A second finding is that all behavioral determinants were mediators in the association between the intervention and fruit intake, apart from social influence. The association of the intervention with fat from bread spread and PA was mediated by action control and the participants’ psychological profile.

An earlier review that concluded there was especially a lack of clear and consistent evidence on effective pathways for dietary behavior, the present study adds knowledge and provides clear evidence on the important contribution of action control to dietary behaviors.

Other studies have also found the mediating role of action control in intervention effectiveness. One study showed the mediating role of action control in changing PA behavior, and another showed this for fruit intake [35, 36]. Action control is seen as a suspect to overcome the intention – behavior gap; meaning an intention to change behavior does not automatically lead to a response in behavior change. Action control is a combination of self-monitoring, awareness and self-regulation, which adds to overcoming obstacles to behavior change. Both earlier studies had an observational design and used formal mediation analyses, therefore our results confirm and strengthen these hypotheses in a randomized design.

All behavioral determinants, except social influence, mediated towards higher fruit intake. This finding can be explained by the fact that fruit intake is a concrete behavior, whereas fat and fiber intake are nutrient values indirectly derived from a number of behavioral actions. Therefore fruit intake may be a behavior that is more easily changed than other dietary behaviors.

No mediating behavioral determinants could be identified for fiber intake from bread, where we could argue that the advice to consume more brown or whole-grain bread shows a discrepancy with fiber content. Regression of types of bread on fibre intake, showed that only whole-grain bread contributed to an increased fiber intake, whereas both white and brown bread intake had an inverse association with increased fiber intake. These findings confirm that dietary advices should focus on whole-grain bread.

Strengths of the present study were that the positive results of the SLIMMER study measured with a randomized study design were combined with formal mediation analyses, allowing us to draw conclusions on the effective pathways of a long-term effective, combined lifestyle intervention in a real-life setting. In addition, using bootstrapping in the mediation analyses makes the inference robust, because bootstrapping is perceived as the superior method in mediation analyses and can handle skewed distributions.

A limitation of the present study is the measurement error associated with measuring behaviors and behavioral determinants. We did use a questionnaire based on validated questionnaires, but measurement tools for these constructs often rely on relative validity by lack of a true gold standard, making it difficult to measure 100% of exposure. This probably explains why the investigated mediating pathways could only explain up to approximately 60% of the intervention effect. However, measurement errors were expected to be equal in both the intervention and the control group, and therefore should not affect our results.

Practical implications of the study are that incorporating combined dietary and PA interventions in real-life settings could help to prevent T2DM in high-risk populations. Especially the finding that increased action control leads to significant lifestyle behavior change is valuable. This implicates that interventions and policies should focus on enclosing the intention – behavior gap, by addressing action control, which means to increase self-monitoring, awareness and self-regulation in individuals. As many policies have relied on the assumption that individuals have sufficient knowledge and skills for making lifestyle and health choices, the assumption should rather be that knowing does not automatically lead to doing, which would be a step towards a different type of health policy. Another of such future options, could also be to adapt environments in order to make healthier choices easier, rather than continually increasing choice options and thereby overchoice.

## Conclusion

In conclusion, the SLIMMER intervention effectively reduced fasting insulin and body weight via both dietary and PA intervention behaviors, through differing pathways. Action control was a consistent mediating behavioral determinant in achieving changes in PA behavior, fruit intake, and fat intake from bread spread. Fruit intake was under more cognitive control than the other health behaviors.

## Additional files

Additional file 1: Items behavioral determinants. (DOCX 116 kb)

Additional file 2: Baseline table behavioral determinants. (DOCX 72 kb)

## Abbreviations

BCTs: Behavior change techniques
DHD index: Dutch healthy diet index
GP: General practitioner
IFG: Impaired fasting glucose
MVPA: Moderate to vigorous-intensity physical activity
NBS index: Nutrition behavior score index
OGTT: Oral glucose tolerance test
PA: Physical activity
SLIMMER: SLIM iMplementation Experience Region Noord- en Oost-Gelderland
SSBs: Sugar-sweetened beverages
