# Supplementary material for: Determinants of lifestyle behavior change to prevent type 2 diabetes in high-risk individuals
Source: Int J Behav Nutr Phys Act. 2017 Jun 12;14:78. doi: 10.1186/s12966-017-0532-9 (PMC5468963; doi:10.1186/s12966-017-0532-9)
Supplement: Supplementary file 1 — Items behavioral determinants. (DOCX 116 kb) [file 12966_2017_532_MOESM1_ESM.docx]

# Additional file 1: Questionnaire items behavioral determinants (in Dutch)

Examples for fruit intake:

### Intention

In hoeverre bent u het met de volgende stellingen eens?

1. Ik ben van plan in de komende maanden minstens 2 stuks fruit per dag te eten
2. Ik verwacht dat ik in de komende maanden minstens 2 stuks fruit per dag zal gaan eten
3. Ik ga proberen de komende maanden minstens 2 stuks fruit per dag te eten

### Attitude

1. [Fijn | Vervelend] Naar mijn mening is het eten van minstens 2 stuks fruit per dag...
2. [Bevredigend | Frustrerend] Naar mijn mening is het eten van minstens 2 stuks fruit per dag...
3. [Goed | Slecht] Naar mijn mening is het eten van minstens 2 stuks fruit per dag...
4. [Belangrijk | Onzin] Naar mijn mening is het eten van minstens 2 stuks fruit per dag...
5. [Wenselijk | Onwenselijk] Naar mijn mening is het eten van minstens 2 stuks fruit per dag...
6. [Makkelijk | Moeilijk] Naar mijn mening is het eten van minstens 2 stuks fruit per dag...

### Social influence

De mensen die heel belangrijk voor me zijn...

1. Vinden het belangrijk dat ik minstens 2 stuks fruit per dag eet
2. Eten minstens 2 stuks fruit per dag
3. Steunen mij als ik minstens 2 stuks fruit per dag wil eten

### Self-efficacy

In hoeverre bent u het met de volgende stellingen eens?

1. Ik ben in staat om minstens 2 stuks fruit per dag te eten onder normale omstandigheden
2. Ik ben in staat om minstens 2 stuks fruit per dag te eten bij speciale gelegenheden (bijv. weekend, feestje of vakantie)
3. Ik ben in staat om minstens 2 stuks fruit per dag te eten als ik druk ben

### Motivation

1. [Erg gemotiveerd | Helemaal niet gemotiveerd] Op een schaal van 1 tot 7, in hoeverre bent u gemotiveerd om minstens 2 stuks fruit per dag te gaan of blijven eten?
2. [Veel vertrouwen | Helemaal geen vertrouwen] Als u zou besluiten om minstens 2 stuks fruit per dag te eten, in hoeverre heeft u er vertrouwen in dat u daarin zult slagen?

### Action control

In hoeverre bent u het eens met de volgende stellingen?

1. Als ik me voorneem om minstens 2 stuks fruit per dag te eten, dan voer ik dat vaak ook uit
2. Ik let altijd goed op of ik wel minstens 2 stuks fruit per dag eet
3. Als ik een dag minder dan 2 stuks fruit eet, probeer ik dat altijd op een andere dag wel te realiseren

### Skills

In hoeverre bent u het eens met de volgende stellingen?

1. Ik kan de etiketten op voedingsmiddelen begrijpen
2. Ik kan de informatie op etiketten van voedingsmiddelen gebruiken om te bepalen wat ik koop
3. Ik kan de informatie op etiketten van voedingsmiddelen gebruiken om te bepalen wat ik eet
4. Ik proef vaak een product dat ik nog nooit gegeten heb
5. Ik kan concrete plannen maken om te gaan bewegen (bijv. waar, wanneer en met wie)Ik kan doelen opstellen om (meer) te gaan bewegen
6. Ik kan afspraken met mezelf maken om gezonder te eten of meer te bewegen
