# Supplementary material for: Determinants of lifestyle behavior change to prevent type 2 diabetes in high-risk individuals
Source: Int J Behav Nutr Phys Act. 2017 Jun 12;14:78. doi: 10.1186/s12966-017-0532-9 (PMC5468963; doi:10.1186/s12966-017-0532-9)
Supplement: Supplementary file 2 — Baseline table behavioral determinants. (DOCX 72 kb) [file 12966_2017_532_MOESM2_ESM.docx]

**Additional table 2: Baseline characteristics of behavioral determinants presented as mean ± SD or n (%) (n = 275)**

| Behavioral determinants: | INT (n=139) | CON (n=136) |
| --- | --- | --- |
| *Intention (scale 1 – 7):* |  |  |
| - Fruit intake | 5.8 ± 1.4 | 5.8 ± 1.3 |
| - Vegetable intake | 6.0 ± 1.1 | 6.0 ± 1.1 |
| - Whole-grain and brown bread intake | 5.6 ± 1.7 | 5.6 ± 1.8 |
| - Lean bread spread intake | 5.8 ± 1.2 | 5.8 ± 1.2 |
| - Healthy snacks | 5.7 ± 1.3 | 5.9 ± 1.0 |
| - SSB intake | 5.7 ± 1.7 | 5.8 ± 1.6 |
| - Physical activity | 5.9 ± 1.1 | 6.0 ± 1.0 |
| *Attitude (scale 1 – 7):* |  |  |
| - Fruit intake | 6.0 ± 1.2 | 6.0 ± 1.2 |
| - Vegetable intake | 6.2 ± 1.0 | 6.1 ± 1.1 |
| - Whole-grain and brown bread intake | 6.4 ± 1.0 | 6.2 ± 1.2 |
| - Lean bread spread intake | 6.0 ± 1.0 | 5.8 ± 1.2 |
| - Healthy snacks | 5.9 ± 1.1 | 5.7 ± 1.1 |
| - SSB intake | 6.1 ± 1.2 | 6.0 ± 1.3 |
| - Physical activity | 6.2 ± 1.0 | 6.2 ± 1.0 |
| *Social influence (scale 1 – 7):* |  |  |
| - Fruit intake | 5.8 ± 1.3 | 5.7 ± 1.5 |
| - Vegetable intake | 6.0 ± 1.2 | 5.8 ± 1.3 |
| - Whole-grain and brown bread intake | 6.1 ± 1.3 | 5.9 ± 1.5 |
| - Lean bread spread intake | 5.8 ± 1.3 | 5.7 ± 1.3 |
| - Healthy snacks | 5.9 ± 1.2 | 5.6 ± 1.3 |
| - SSB intake | 5.7 ± 1.5 | 5.5 ± 1.3 |
| - Physical activity | 5.8 ± 1.3 | 5.8 ± 1.3 |
| *Self-efficacy (scale 1 – 7):* |  |  |
| - Fruit intake | 5.9 ± 1.2 | 6.0 ± 1.0 |
| - Vegetable intake | 5.9 ± 1.1 | 5.9 ± 1.0 |
| - Whole-grain and brown bread intake | 5.9 ± 1.4 | 6.0 ± 1.3 |
| - Lean bread spread intake | 5.8 ± 1.0 | 5.8 ± 1.0 |
| - Healthy snacks | 5.6 ± 1.0 | 5.6 ± 0.9 |
| - SSB intake | 5.9 ± 1.4 | 6.2 ± 1.0 |
| - Physical activity | 5.7 ± 1.1 | 5.7 ± 1.2 |
| *Motivation (scale 1 – 7):* |  |  |
| - Fruit intake | 5.6 ± 1.8 | 5.7 ± 1.6 |
| - Vegetable intake | 5.7 ± 1.6 | 5.7 ± 1.7 |
| - Whole-grain and brown bread intake | 6.0 ± 1.7 | 5.8 ± 1.9 |
| - Lean bread spread intake | 5.6 ± 1.5 | 5.5 ± 1.5 |
| - Healthy snacks | 5.5 ± 1.4 | 5.5 ± 1.4 |
| - SSB intake | 6.0 ± 1.6 | 6.0 ± 1.6 |
| - Physical activity | 5.8 ± 1.4 | 5.7 ± 1.4 |
| *Action control (scale 1 – 7):* |  |  |
| - Fruit intake | 5.7 ± 1.4 | 5.7 ± 1.2 |
| - Vegetable intake | 5.9 ± 1.3 | 5.7 ± 1.2 |
| - Whole-grain and brown bread intake | 6.1 ± 1.2 | 6.0 ± 1.3 |
| - Lean bread spread intake | 5.8 ± 1.1 | 5.5 ± 1.2 |
| - Healthy snacks | 5.5 ± 1.2 | 5.4 ± 1.2 |
| - SSB intake | 5.9 ± 1.3 | 5.9 ± 1.2 |
| - Physical activity | 5.5 ± 1.3 | 5.5 ± 1.2 |
| *Skills (scale 1 – 7):* |  |  |
| - Dietary | 5.4 ± 0.8 | 5.2 ± 1.1 |
| - Physical activity | 5.5 ± 1.2 | 5.5 ± 1.2 |
